# Crystallization of Galectin-8 Linker Reveals Intricate Relationship between the N-terminal Tail and the Linker

**DOI:** 10.3390/ijms17122088

**Published:** 2016-12-12

**Authors:** Yunlong Si, Yue Wang, Jin Gao, Chenyang Song, Shiqiong Feng, Yifa Zhou, Guihua Tai, Jiyong Su

**Affiliations:** Jilin Province Key Laboratory on Chemistry and Biology of Natural Drugs in Changbai Mountain, School of Life Sciences, Northeast Normal University, Changchun 130024, China; siyl071@nenu.edu.cn (Y.S.); wangy147@nenu.edu.cn (Y.W.); gaoj466@nenu.edu.cn (J.G.); songcy738@nenu.edu.cn (C.S.); fengsq104@nenu.edu.cn (S.F.); zhouyf383@nenu.edu.cn (Y.Z.); taigh477@nenu.edu.cn (G.T.)

**Keywords:** galectin-8 N-terminal CRD crystal structure, galectin linker crystallization, hemagglutination assay, glycerol

## Abstract

Galectin-8 (Gal-8) plays a significant role in normal immunological function as well as in cancer. This lectin contains two carbohydrate recognition domains (CRD) connected by a peptide linker. The N-terminal CRD determines ligand binding specificity, whereas the linker has been proposed to regulate overall Gal-8 function, including multimerization and biological activity. Here, we crystallized the Gal-8 N-terminal CRD with the peptide linker using a crystallization condition that contains Ni^2+^. The Ni^2+^ ion was found to be complexed between two CRDs via crystal packing contacts. The coordination between Ni^2+^ and Asp25 plays an indirect role in determining the structure of β-strand F0 and in influencing the linker conformation which could not be defined due to its dynamic nature. The linker was also shortened in situ and crystallized under a different condition, leading to a higher resolution structure refined to 1.08 Å. This crystal structure allowed definition of a short portion of the linker interacting with the Gal-8 N-terminal tail via ionic interactions and hydrogen bonds. Observation of two Gal-8 N-terminal CRD structures implies that the N-terminal tail and the linker may influence each other’s conformation. In addition, under specific crystallization conditions, glycerol could replace lactose and was observed at the carbohydrate binding site. However, glycerol did not show inhibition activity in hemagglutination assay.

## 1. Introduction

Galectins are a family of proteins with some specificity for β-galactoside binding at their carbohydrate-recognition domain (CRD) [1,2,3,4]. Eleven galectins (Galectin-1, -2, -3, -4, -7, -8, -9, -10, -12, -13, and -14) have been identified so far in the human genome [3,5]. These are classified as prototype, chimeric type, and tandem-repeat-type [5,6,7,8]. Tandem-repeat-type galectins, like Gal-8, contain two distinct CRDs that are covalently connected via a peptide linker [5,6,7,8,9]. Gal-8 was initially cloned from a library of rat liver cDNA that contains an open reading frame encoding for 317 amino acids (~35 kDa) [10]. Biological studies with Gal-8 have revealed that it can both positively and negatively regulate cell adhesion, depending on its extracellular concentration [11,12]. A case-control analysis and related quantitative trait-association study indicated that the point mutation F19Y is associated with rheumatoid arthritis [13].

The transcription and translation of Gal-8 in different tissues were examined by Northern and Western blot analyses [10,11,14]. These results demonstrated that Gal-8 is expressed in many tissues, including lung, liver, kidney, spleen, hind-limb, and cardiac muscle. Low levels of expression were also detected in the intestine, colon, fat, and thymus. The Gal-8 gene (*LGALS8*) was found to encode seven mRNAs by alternate splicing. These mRNAs encode six different isoforms of Gal-8. Three isoforms belong to the tandem-repeat galectin group (with two CRDs) and the others to the prototype group (with one CRD). The tandem-repeat isoforms differ in the length of their linker peptide [15,16]. Based on the size of the linker, Gal-8 tandem-repeat isoforms have been classified into Gal-8S (small gal-8, with a short linker), Gal-8M (medium gal-8, with an intermediate linker, the ‘canonical’ sequence), and Gal-8L (long gal-8, with the longest linker) [15,16]. The linker was found to play an important role in regulating Gal-8 function. The deletion of the linker severely impairs Gal-8 in mediating cell adhesion [17]. However, a protease-resistant mutant of Gal-8 [18] that lacks the entire linker (Gal8M) exhibits similar in vitro biological activity [19]. Until now, however, the conformation of the linker has not been defined. Given its importance, it has become a high priority to solve the structure of Gal-8 with at least some of the linker in place, as well as to further investigate the physiological function of this flexible domain.

The affinity of Gal-8 for various ligands has been assessed using frontal affinity chromatography, revealing that this lectin has a particular preference for some glycolipid-type glycans, binding most strongly to acidic saccharides. Further analysis of these results showed that this binding preference is attributable to its N-terminal CRD [20]. ELISA and surface plasmon resonance assays were also used to determine the affinity between Gal-8 and some glycoconjugates. These results demonstrated that Gal-8 has a much higher affinity for 3′-O-sulfated or 3′-O-sialylated glycoconjugates and a Lewis X-containing glycan rather than oligosaccharides that terminate in Galβ1 → 3/4GlcNAc. This specificity is also attributed to the N-terminal CRD [21]. Moreover, co-crystal structures of the Gal-8 N-terminal CRD with sulphated/sialylated oligosaccharides showed that Arg45, Gln47, Arg59, and the long loop between S3 and S4 β-strands directly or indirectly interact with sulfate or sialic acid moieties [22]. Due to the importance of the Gal8 N CRD, we have investigated its crystal structure and additional ligand binding properties.

Residues in the carbohydrate binding site are generally conserved in the CRD of all galectins; therefore, all galectins interact with β-galactosides. However, the carbohydrate binding site in Gal-3 and Gal-4 can also accommodate glycerol. In the glycerol-loaded co-crystal structures of Gal-3 and Gal-4, the three carbon and three oxygen atoms of glycerol occupy the same positions as C4, C5, C6 and O1, O4, O6 of galactose [23,24,25]. Another structure of glycerol-loaded Gal-3 showed that two molecules of glycerol could fit into the carbohydrate binding site. The oxygen and carbon atoms of the first glycerol are positioned identically to galactose atoms O4-O6 and C4-C6, whereas the second glycerol molecule was found in the same position as the O1, O3′, O5′, and C3′-C5′ atoms of glucose [24]. Glycerol is a flexible molecule and can adopt many conformations in water [26]. NMR studies demonstrated that the carbohydrate binding site of Gal-3 is flexible in the absence of any ligand, indicating that flexibility on both sides plays a role in interactions between glycerol and the CRD [27,28].

In the present study, we used different conditions to crystallize the Gal-8 N-terminal CRD with its linker, and five structures could be solved. SDS-PAGE indicated that one of these preparations maintained the entire linker, because the molecule weight of the Gal-8 N-terminal CRD/linker was the same before and after crystallization. Under another crystallization condition, the linker was found to be shortened. One crystal from this condition displayed an atomic resolution of 1.08 Å, the highest resolution structure of Gal-8 N-terminal CRD available to date. Interestingly, glycerol was found to substitute lactose at the carbohydrate binding site in 1.08 Å structure, however, because the hemagglutination assay showed that glycerol did not inhibit Gal-8 N-mediated agglutination, this ligand could not be physiologically relevant.

## 2. Results

### 2.1. Gal-8_1-186 Induces Erythrocyte Agglutination

**Figure 1:**
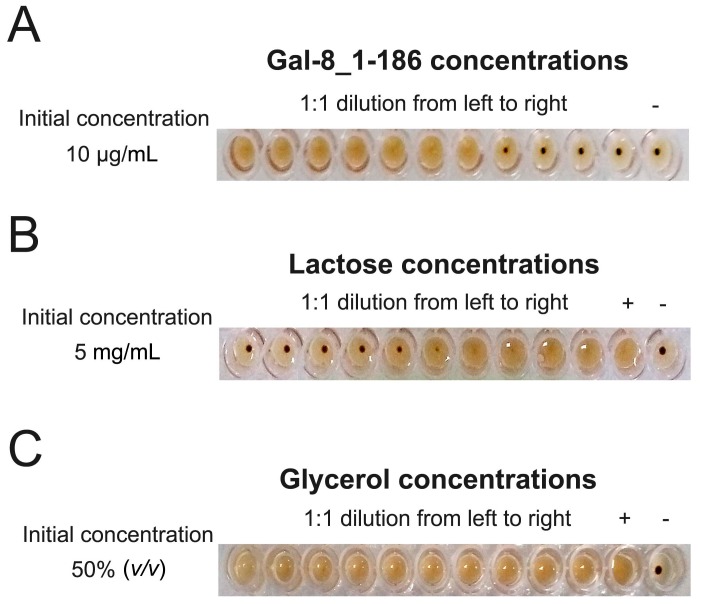
Gal-8_1-186 induces chicken erythrocyte agglutination assay. (**A**) Gal-8_1-186 induce agglutination of chicken erythrocytes with a MAC (Minimum Agglutination Concentration) value of 0.16 µg/mL. “−”represents the negative control well containing no protein; (**B**) the agglutination capability of Gal-8_1-186 was inhibited by lactose. 0.16 µg/mL Gal-8_1-186 was used in this assay. The MIC (Minimum Inhibition Concentration) of lactose is 0.63 mg/mL. “−”represents the negative control well containing no protein and lactose; “+”represents the positive control well containing no lactose; (**C**) the agglutination capability of Gal-8_1-186 was inhibited by glycerol. 0.16 µg/mL Gal-8_1-186 was used in this assay. Glycerol cannot inhibit the agglutination activity of Gal-8_1-186 even at 50% (*v*/*v*) concentrations. “−” represents the negative control well containing no protein and glycerol; “+” represents the positive control well containing no glycerol.

The hemagglutination assay shows that truncated Gal-8_1-186 induces agglutination of chicken erythrocytes with a Minimum Agglutination Concentration (MAC) of 0.16 µg/mL (Figure 1A). Even though this truncated variant of Gal-8 with only one CRD could induce agglutination, previous chemical cross-linking studies did show that it could form dimers [29], and one crystal structure did show that the Gal-8 N CRD did form dimers via crystal packing contacts [22]. This suggests that our truncated Gal-8 may induce erythrocyte agglutination by forming some type of dimeric structure. Furthermore, lactose did inhibit the biological activity of Gal-8_1-186, with a Minimum Inhibition Concentration (MIC) around 0.63 mg/mL for the disaccharide (Figure 1B). Because one of our structures reported here did bind glycerol (see the following section), we investigated whether glycerol could inhibit Gal-8 N-induced agglutination. Our results demonstrate that glycerol could not inhibit Gal-8 biological activity, indicating, as expected, that glycerol is not a physiological ligand of Gal-8 (Figure 1C), consistent with a previous report on glycerol and Gal-3 [24].

### 2.2. SDS-PAGE of Protein Crystals

**Figure 2:**
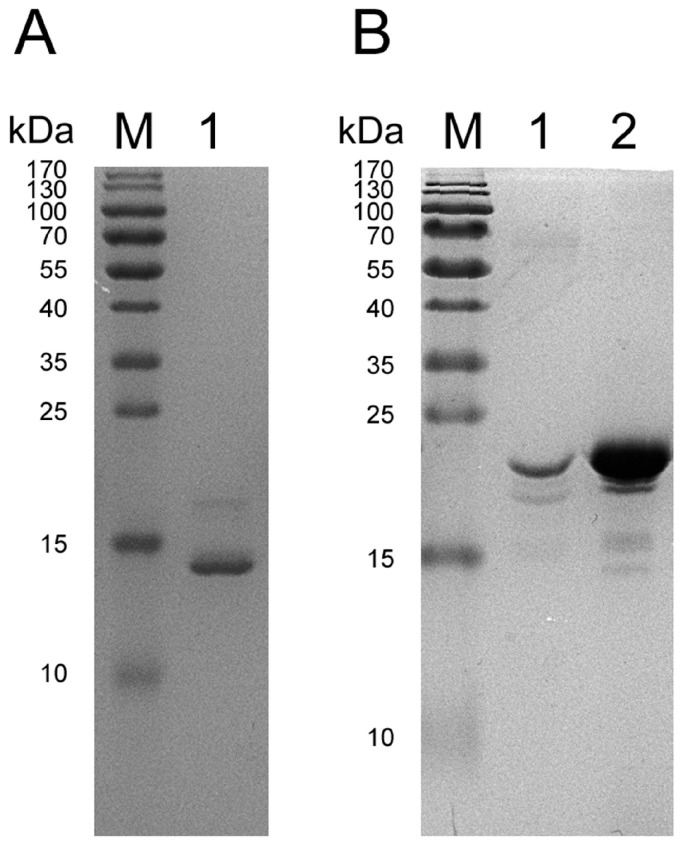
SDS-PAGEs of protein crystals. (**A**) “M” indicates protein molecular marker; “1” indicates the crystallized protein in the first crystallization condition containing 20% (*w*/*v*) PEG2000 MME. The molecular weight of this protein is approximately 14 kDa; (**B**) “M” indicates protein molecular marker; “1” indicates the crystallized protein in the second crystallization condition containing 0.1 M Tris pH 8.5, 0.01 M NiCl_2_, 20% (*w*/*v*) PEG2000 MME; “2” indicates the protein after his-tag cut off. The protein bands in “1” and ”2” are same that means Gal-8 N-terminal CRD with the linker was crystallized.

SDS-PAGE of re-dissolved protein crystals from two crystallization conditions demonstrated that the molecular weights of the crystallized proteins are different (Figure 2). Prior to crystallization trials, the His-tag was removed from the truncated protein by thrombin digestion (i.e., at VPR|GSHM) over 24 h, but the protease was not separated from the Gal-8_1-186 preparation. In this regard, Figure 2A indicates that thrombin may slowly digest Gal-8_1-186, reducing its size. The smaller fragment, which contains the N-terminal CRD and a short piece of the linker, was then crystallized. This shorter variant produced crystals yielding a higher resolution structure (Table 1).

In Figure 2B, the molecular weight of the crystallized protein was found to be the same before and after crystallization, indicating that the Gal-8 N-terminal CRD with the linker was crystallized. Unfortunately, the structure of the linker could not be defined, because following crystal structure refinement, no electron density for the linker was observed (from Ser154 to end), indicating that the linker is highly flexible.

### 2.3. Description of the Gal-8_1-186 Structures

The five refined structures (structures 1–5, PDB codes are 5GZC, 5GZD, 5GZE, 5GZF, 5GZG) for these Gal-8 N crystals reported here (Table 1) are compared with previously reported crystal structures [30,31]. Superimposition of all of these structures yielded Cα r.m.s. deviations of 0.1955 Å to 0.8470 Å. Our five structures were solved from crystals grown under two different conditions: (1) 20% (*w*/*v*) PEG2000 MME and (2) 0.1 M Tris pH 8.5, 0.01 M NiCl_2_, 20% (*w*/*v*) PEG2000 MME. Structures 1–3 were solved from the first condition and cryoprotectants for these three structures were glycerol, ethylglycol, and PEG400, respectively. Structures 4 and 5 were solved from the second condition and cryoprotectants for these two structures were glycerol and ethylglycol, respectively.

**Figure 3:**
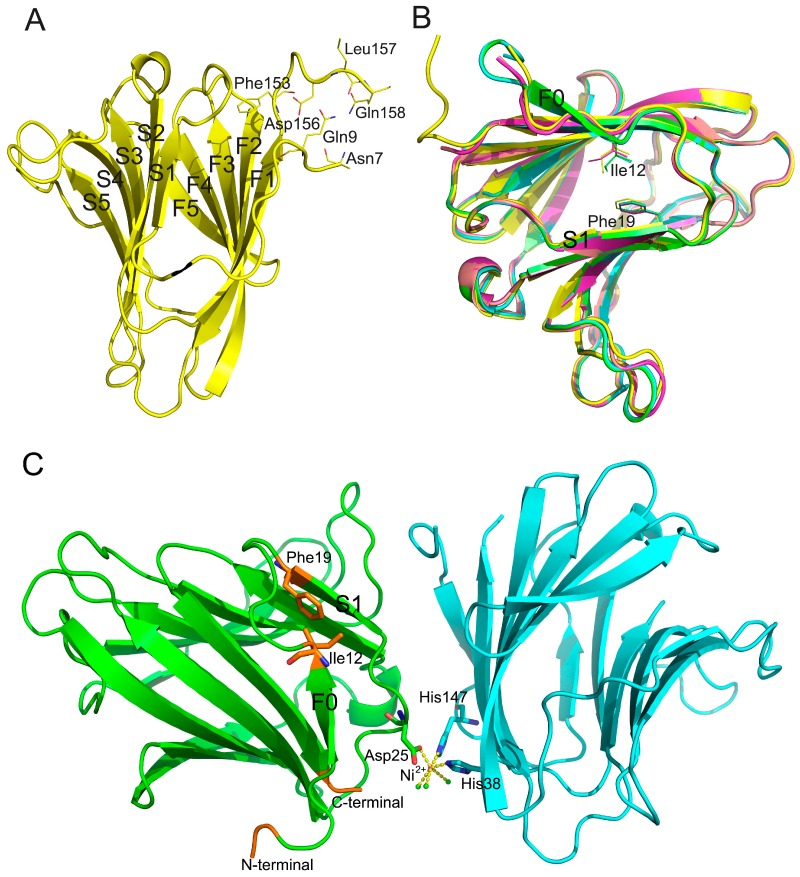
Crystal structures of Gal-8_1-186. (**A**) The N-terminal tail interacts with the bottom of Gal-8 linker (structure 1); (**B**) Phe19 could interact with Ile12 through van der Waals forces (structure 1: **yellow**; structure 2: **purple**; structure 3: **pink**; structure 4: **cyan**; structure 5: **green**); (**C**) Ni^2+^ was found to form a complex with Asp25 and two histidine residues (His38 and His147) from another Gal-8N molecule. Two Gal-8 N-terminal CRDs from two asymmetric units were labeled by green and cyan. This figure was generated by using structure 4 (PDB: 5GZF). One pink ball indicates Ni^2+^. Three green balls indicate water molecules. N-terminal and C-terminal of the structure are close to each other and are labeled by gold. C-terminal of the structure is also the starting point of the linker.

The best structure, structure 1, allowed 152 residues (Asn7 to Gln158) to be identified with 1.08 Å resolution. This is the best resolution for the Gal-8 N CRD available to date. The initial six residues (Met1 to Asn6) at the N-terminus of the CRD were not visible, likely due to structural disorder within the crystals. Electron density of the first four residues in the linker region (Ser154 to Gln158) was clear, indicating that this part of linker forms stable secondary structure under conditions of our crystallization. In structure 1, four residues (Asn7, Gln9, Asp156, and Gln158) were seen to interact with each other via ionic interactions and hydrogen bonds (Figure 3A). Although the crystallization conditions were the same for structures 2 and 3, residues from the linker were less visible compared to our 1.08 Å structure. In structures 4 and 5, the electron density of residues from Ser154 to the C-terminus was not visible, suggesting greater flexibility from this segment. In structures 1–3, the N terminal tail (Gln9 to Ile12) forms a rigid loop (Figure 3B), whereas in structures 4 and 5, the N terminal tail (Gln9 to Ile12) forms a small β-strand F0.

In structures 4 and 5, Ni^2+^ formed a complex with Asp25 and His38, His147 from another Gal-8N molecule, along with three water molecules (Figure 3C). In contrast in structures 1 to 3, no divalent caution was observed at this position. In a previously reported structure, Zn^2+^ was found at the same position as Ni^2+^ in the crystal structure with space group *P*4_3_2_1_2 [32]. Asp25 is close to β-strand S1, and coordination between Asp25 and Ni^2+^ may impact on S1. Therefore, S1 may induce Gln9 to Ile12 to form a β-strand from Ile12 to Phe19 via van der Waals forces.

It appears that there is a relationship between the N-terminal tail (especially Gln9 to Ile12 region) and the linker. If the N-terminal tail interacts with the linker, then Gln9 to Ile12 forms a rigid loop and part of the linker (here is from Ser154 to Gln158) is stabilized (Figure 3A). In contrast, if the N-terminal tail cannot interact with the linker for some reason (e.g., due to the Ni^2+^ ion mentioned above), then Gln9 to Ile12 forms a β-strand with the linker having high flexibility. This would indicate that the N-terminal tail and the linker could influence the conformation of each other.

Gal-8 variant F19Y is significantly associated with rheumatoid arthritis [13]. If Phe19 were mutated to Tyr, then the hydroxyl group of Tyr could influence the interaction between Phe19 and Ile12, such that the structure of the N-terminal tail would be influenced. After that, the structure of linker should also be changed. Finally, alternation of linker conformation could also result in global effects to the Gal-8 structure and, therefore, function. This prospect is consistent with the previously published results from circular dichroism (CD) [32]. There, a far-UV CD spectrum revealed a slight secondary structure difference between Gal-8 N-terminal CRD and the F19Y variant. [32]. The CD spectrum of thermal denaturation experiments also showed that the T_1/2_ of the full-length form of the F19Y variant was 2.5 °C higher than that of the wild type protein. This indicates that the impact from the F19Y substitution on the structure of the lectin is not confined to the N-terminal domain [32].

### 2.4. Effect of Cryoprotectants on Gal-8_1-186 Crystals

**Figure 4:**
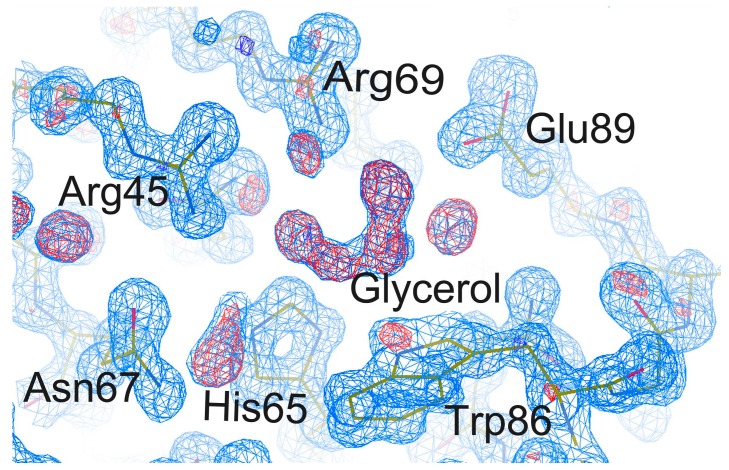
Electron density map of the carbohydrate binding site binds to glycerol in structure 1 (PDB code: 5GZC) of Gal-8_1-186. The 2|*F_o_*| − |*F_c_*|, αc map contoured at 1δ is shown as blue density and the |*F_o_*| − |*F_c_*|, αc map contoured at 3δ is shown as red density.

Surprisingly, crystal structure 1 made using the first condition has space group *P*2_1_2_1_2_1_, and not *C*2, and the carbohydrate binding site binds glycerol and not lactose (Figure 4). This structure was solved with the crystal being soaked in 20% (*v*/*v*) glycerol for approximately 1 min prior to data collection. This indicates that using this high concentration of glycerol changed the space group of crystal from *C*2 to *P*2_1_2_1_2_1_, while replacing lactose at the carbohydrate binding site. The resolution of this structure is 1.08 Å, which is higher than any other Gal-8 structure solved to date. When using XDS or Aimless, the geometric parameters of our five structures could not be modified using different geometric parameters.

Glycerol can be used as a cryoprotectant in cryoannealing, a technique used to improve the quality of crystal data sets and minimize *R* values. After several cycles of flash-cooling and room temperature exposure, the quality of crystals presumed to be better [33]. In addition, the geometric parameters of several crystals could be changed by using this technique. Before the Gal-8 N crystal was loaded onto the data collection unit, the crystal was exposed to room temperature for approximately 2 s. Even though this approach is different from cryoannealing, it may accomplish the same result. However, the presence of glycerol did not influence the geometric parameters of the structures from crystals made using the second crystallization condition (Table 1). This indicates that the crystal packing contacts in the *P*4_3_2_1_2 structure were stable.

Glycerol, ethylene glycol, and PEG400 are usually used as cryoprotectants for production of galectin crystals. Here, we compared the effects of cryoprotectants on the Gal-8 N CRD structures. The resolution of *C*2 structures (structures 2 and 3, cryo-protected by ethylene glycol and PEG400, respectively) are lower than our *P*2_1_2_1_2_1_ structure (structure 1, cryo-protected by glycerol). The *R*_free_ values of the *C*2 structures are higher than our *P*2_1_2_1_2_1_ structure, especially the *R*_free_ value of the PEG400 structure. This indicates that cryoprotectants do play a role in determining the quality of Gal-8 N crystal structures.

### 2.5. The Carbohydrate Binding Site

**Figure 5:**
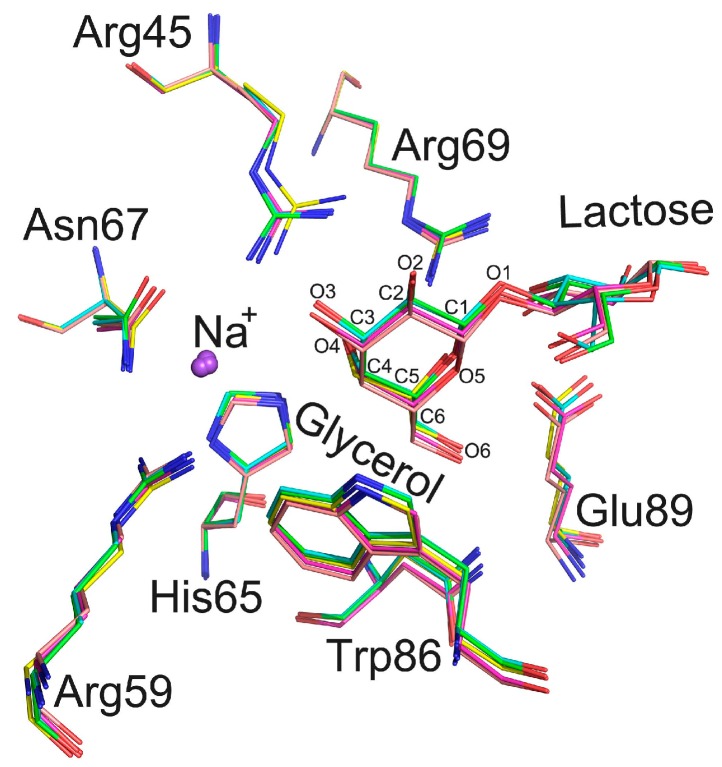
The carbohydrate binding sites of Gal-8_1-186 of five structures are merged. Nitrogen atoms are labeled by blue. Carbon atoms are labeled by red. The covalent bonds from structures 1–5 are labeled by yellow, purple, pink, cyan and green, respectively. The positions of residues (Arg45, His65, Asn67, Arg69, Trp86, and Glu89) could coordinate with glycerol and lactose. The C and O atoms of glycerol occupy the same sites as C4, C5, C6 and O1, O4, O6 of galactose. A Na^+^ ion, observed in Gal-8_1-186 structures, is close to the carbohydrate binding site.

In structure 1, glycerol was found to replace lactose in the carbohydrate binding site. The positions of residues Arg45, His65, Asn67, Arg69, Trp86, and Glu89 coordinate with both glycerol and lactose (Figure 5). However, the side chain of Arg45 in this structure shifts by approximately 0.5 Å compared to that in other structures. In structures of Gal-3, an Arg residue is also located at the same position as Arg 162. This Arg residue has been suggested to coordinate the pyranose ring of lactose in the Gal-3 structure. It seems that this arginine in the Gal-8N CRD can adopt different conformations depending upon which ligand binds to the Gal-8 N CRD.

In our crystal structure, the C and O atoms of glycerol occupy the same positions as C4, C5, C6 and O1, O4, O6 atoms of galactose in the lactose-bound structure (Figure 5). Wild type Gal-8 N 1-152 has been crystallized in the *P*4_3_2_1_2 space group [13] when glycerol was also used as the cryoprotectant. However, in that study, glycerol did not replace lactose, and was found to be located close to Tyr141 [13]. Moreover, in that instance, glycerol did not influence lactose binding to the Gal-8 N-terminal CRD. In our *P*4_3_2_1_2 structure, glycerol also could not replace lactose. Taken together, this likely indicates that either the binding affinity between lactose and the Gal-8 N CRD is different under different crystallization conditions or that the ability of glycerol to replace lactose in the carbohydrate binding site is different under different crystallization conditions. Under solution conditions used in the hemagglutination assay, Gal-8_1-186 may not have high affinity for glycans on erythrocyte membranes. On the other hand, glycerol could not inhibit erythrocyte agglutination induced by Gal-8_1-186. Furthermore, the Gal-8 N-terminal CRD in solid (crystallized) and liquid states may have different affinities towards various ligands. Therefore, glycerol could replace lactose in the Gal-8 N-terminal CRD in the crystallized form, and could not replace competition with carbohydrates in the agglutination assay.

Collins et al. reported that the time scale of glycerol displacement for lactose in Gal-3 crystals is on the order of days [23]. Glycerol is a weak ligand for Gal-3 [24], such that high affinity ligands should readily displace glycerol. This approach has been suggested as a good method to exchange ligands with Gal-3. Here, we observed that the lactose displacement time with glycerol in Gal-8 N-terminal CRD crystals was only ~1 min, much shorter than that found with Gal-3. This suggests that with Gal-8 N, it is feasible to use this approach for ligand exchange.

## 3. Discussion

Human tandem-repeat-type lectins (Gal-4, -8, -9, and -12) contain two CRDs, whose structures are highly homologous. However, the linker connecting their N- and C-CRDs, as well as the short tail preceding the N-CRD, is highly variable. This variance likely determines a given lectins’ specific functional role. For example, these linkers play roles in interactions with other proteins, membrane insertions, and the regulation of CRD presentations. On the other hand, these diverse linkers are also involved in strengthening signaling potency by intermolecular interactions between CRDs and through multimerization that increases lattice formation on the cell surface [34]. However, the conformation of these linkers remains unknown due likely to their high flexibility. Here, we have successfully crystallized the Gal-8 N-terminal CRD with its linker under crystallization conditions that contain 0.01 M Ni^2+^, a condition that is known to induce trimerization of Gal-9 CRDs [35]. Although the structure of the complete linker was not determined in our structures, the information that we did uncover may provide clues to explain the functions of Gal-8.

In this study, we did not separate thrombin from Gal-8_1-186, primarily because the protease may play a role in Gal-8 L function. In a previous report with Gal-8L, thrombin could digest Gal-8 L, but not Gal-8 M [36]. In that study, however, the digestion time was shorter than the crystallization time (at least two weeks) used here, and the digestion site was assumed to be Arg-Gly and Arg-Thr in the linker region of Gal-8 L. Here, we based the Gal-8 truncation of the linker on Gal-8 M, where the linker region does not contain the RG and RT recognition element. However, thrombin may still have digested our truncated protein. Actually, thrombin is not a digestion site-specific protease, so it could digest some sites non-specifically [37]. Long incubation times with thrombin could explain why only part of the linker was identified using our condition 1 (i.e., 20% (*w*/*v*) PEG2000 MME). Nevertheless, we could observe that this part of the linker did interact with the N-terminus of Gal-8 N via hydrogen bond formation. This is consistent with the predicted model of Gal-8, which suggests that the linker can sometimes form a secondary structure [38].

Frontal affinity gel chromatography [20] and fluorescence polarization have shown that the ligand binding specificities of the two Gal-8 CRDs are different. The N-terminal CRD prefers sulfated and sialylated glycans, whereas the C-terminal CRD prefers blood group A/B [39]. In particular, the N-terminal CRD strongly binds to sulfated and sialylated glycans. The crystal structure of the N-terminal CRD bound with sulfated and sialylated glycans has been solved [22], and it has been suggested that the N-terminal CRD determines the function of Gal-8 [21]. In this present report, one of our structures shows that glycerol binds to the carbohydrate binding site. Therefore, it may be a good idea to substitute lactose in Gal-8 N CRD crystals with glycerol and then replace glycerol with a high affinity ligand.

## 4. Materials and Methods

### 4.1. Cloning, Expression, and Purification of Gal-8_1-186

The Gal-8 (residues 1-317, uniprot identifier: O00214-1) gene was artificially synthesized by SynBio Technologies (Monmouth Junction, NJ, USA). Gal-8_1-186 was amplified using the following primers, Gal-8_1-186 forward: 5′-GGATCCCATATGATGCTGAGCCTGAAC-3′, Gal-8_1-186 reverse: 3′-GGATCCCTCGAGTTACGGCAGACGCAGTTG-5′, which contain *NdeI* and *XhoI* restriction sites. PCR products were digested with *NdeI* and *XhoI* and subsequently cloned into a pET28a vector (Novagen, Gibbstown, NJ, USA). His-tag was constructed in the N-terminal of Gal-8_1-186. His-tagged Gal-8_1-186 containing the N-terminal tail (1–18), N-CRD (19–152) and linker (153–186) was constructed into the pET28a vector. All constructs were confirmed by DNA sequencing. *Escherichia coli* BL21 (DE3) cells were transformed with this construct and induced to express proteins by incubating with 0.5 mM IPTG for 16 h at 25 °C. The protein was extracted and purified using a Ni-NTA agarose column (Qiagen, Hilden, Germany) according to previously reported protocols [40,41]. Following purification, Gal-8_1-186 protein was dialyzed in 10 mM Tris/HCl pH 8.0, 150 mM NaCl, and 10 mM lactose. During overnight dialysis at 4 °C, thrombin was added to remove His tags from the Gal-8_1-186 protein. Each mg of His-tagged protein was digested with 10 units (NIH unit) of thrombin. As determined by sodium dodecyl sulfate-polyacrylamide gel electrophoresis (SDS-PAGE), the purity of the resulting protein was >90%. Finally, proteins were concentrated to 20 mg/mL using an Amicon Ultra-15 Centrifugal Filter Unit (3 kDa cut off) and stored at −80 °C until use. Purification on the His-tag affinity column resulted in a yield of 15 mg protein from 1 L *E. coli* culture.

### 4.2. Crystallization, Data Collection, and Structure Determination

Hampton Research (Aliso Viejo, CA, USA) packs (PEGRx1, PEGRx2, Index, Crystal Screen, and Crystal Screen 2) were used for the initial crystallization screen (sitting-drop vapor diffusion method). After four weeks of incubation at 25 °C, small crystals had formed under the conditions of Crystal screen 2 No. 45 (0.1 M Tris pH 8.5, 0.01 M NiCl_2_, 20% (*w*/*v*) PEG2000 MME). To obtain a crystal that was suitable for X-ray diffraction, we used the hanging-drop and sitting-drop methods. Larger crystals were obtained after about two weeks from drops that contained 1 μL protein (12 mg/mL) and 1 μL solution from the well containing 20% (*w*/*v*) PEG2000 MME (hanging-drop method) or 0.1 M Tris pH 8.5, 0.01 M NiCl_2_, 20% (*w*/*v*) PEG2000 MME (sitting-drop method) at 25 °C.

The crystals were fished out from the crystallization drops and were washed by the reservoir solution. The crystallized proteins were identified by SDS-PAGE with coomassie blue staining.

Prior to X-ray data collection, crystals were soaked for approximately 1 min in the reservoir solution supplemented with 20% (*v*/*v*) glycerol, etheglycol or PEG400 as a cryoprotectant, and then flash cooled in liquid nitrogen. All crystals were grown at the same temperature. Data sets were collected at 100 K at the Shanghai Synchrotron Radiation Facility (Shanghai, China).

Data sets were indexed and integrated using XDS (MPI for Medical Research, Heidelberg, Germany) [42] and scaled using Aimless [43] from the CCP4 package (6.4.0, Science and Technology Facilities Council, Rutherford Appleton Laboratory, Didcot, Oxon, UK, 2015) [44]. Structures were determined by Phaser (University of Cambridge, Cambridge, UK) [45] with a molecular replacement method using the structure of Gal-8 variant (PDB: 4BME) as the search model. Structure refinement and water updating were performed using Phenix (Lawrence Berkeley Laboratory, Berkeley, CA, USA) [46] refine and manual adjustment. Final structure validations were performed using MolProbity (Duke University, Durham, NC, USA) [47,48]. Figures for all structures were generated using PyMOL (Schrödinger K.K, Cambridge, MA, USA) [49] or Coot (MRC Laboratory of Molecular Biology, Cambridge, UK) [50].

### 4.3. Hemagglutination Assay

The hemagglutination assay was performed according to Gao et al. [51]. With some minor modifications. Chicken erythrocytes were prepared from fresh blood collected in Alsever’s medium (2.05% glucose, 0.8% sodium citrate, 0.42% sodium chloride, and 0.055% citric acid) and were washed four times with 0.15 M NaCl. Cells were then suspended in 4% (*v*/*v*) PBS (pH 7.4) containing 1 mg/mL trypsin and incubated at 37 °C for 1 h. After washing with 0.15 M NaCl, the cells were fixed for 1 h in PBS (pH 7.4) containing 1% glutaraldehyde at room temperature followed by termination with five volumes of 0.1 M glycine in PBS (pH 7.4). The fixed cells were washed and adjusted to 10% (*v*/*v*) with PBS (pH 7.4). Cells were maintained at 4 °C until use. The hemagglutination assay was performed in microtiter V plates, with each well containing Gal-8_1-186 in 75 μL Tris buffer (10 mM Tris-HCl, 150 mM NaCl, pH 8.0) or 75 μL Tris buffer and 25 μL 4% (*v*/*v*) chicken erythrocyte suspensions. The cells were added last, followed by shaking, and agglutination was allowed to proceed for 60 min on the ice to ensure the temperature was consistent. Lactose and glycerol were used to inhibit the biological activity of Gal-8_1-186 protein.

## Figures and Tables

**Table 1:** Crystallographic data collection and refinement statistics ^a^.

Structure Name	Structure 1	Structure 2	Structure 3	Structure 4	Structure 5
PDB code	5GZC	5GZD	5GZE	5GZF	5GZG
Resolution (Å)	20.00–1.08 (1.10–1.08)	20.00–1.19 (1.21–1.19)	20.00–1.32 (1.35–1.32)	20.00–2.00 (2.05–2.00)	20.00–2.00 (2.03–2.00)
Space group	*P*2_1_2_1_2_1_	*C*2	*C*2	*P*4_3_2_1_2	*P*4_3_2_1_2
Unit cell parameters (α, β, γ, a, b, c) (Å)	90, 90, 90, 40.56, 49.38, 76.68	90, 108.93, 90, 60.53, 40.05, 59.18	90, 108.02, 90, 60.69, 39.74, 55.80	90, 90, 90, 48.87, 48.87, 159.73	90, 90, 90, 49.13, 49.13, 159.67
no. of measured reflections	776,761 (19,377)	258,417 (10,225)	187,310 (5330)	847,50 (6299)	851,78 (5364)
no. of unique reflections	65,391 (3047)	40,642 (1864)	29,391 (1205)	13,850 (1003)	14,014 (978)
Completeness (%)	98.0 (93.1)	99.3 (89.9)	98.2 (80.2)	99.7 (98.4)	99.3 (95.1)
multiplicity	11.9 (6.4)	6.4 (5.5)	6.4 (4.4)	6.1 (6.3)	6.1 (5.5)
*R*_merge_ (%) ^b^	9.9 (72.7)	4.6 (31.8)	7.2 (49.2)	9.8 (22.5)	5.6 (70.7)
<I/∑(I)>	10.9 (2.4)	17.5 (4.2)	13.4 (2.1)	13.3 (6.7)	14.9 (1.9)
Refine					
Resolution limits (Å)	20.00–1.08	20.00–1.19	20.00–1.32	20.00–2.00	20.00–2.00
*R*_model_ (%) ^c^	15.67	20.55	25.22	19.80	20.27
*R*_free_ (%) ^d^	16.27	21.39	28.08	21.52	25.80
Rmsd bond lengths (Å)	0.008	0.01	0.011	0.008	0.007
Rmsd bond angles (°)	1.047	1.398	1.422	0.993	1.062
Ramachandran plot ^e^ residue in favored regions (%)	99.3%	98.6%	95.9%	97.3%	96.6%
Ramachandran outliers (%)	1.5	0	0	0.8	0.8

^a^ Values in parentheses correspond to the highest-resolution shell; ^b^ *R*_merge_ = ∑_hkl_∑_i_|I(hkl)_i_ − I(hkl)|/∑_hkl_∑_i_I(hkl)_I_; ^c^ *R*_model_ = ∑_hkl_|F_o_(hkl) − F_c_(hkl)|/∑_hkl_ |F_o_(hkl)|, where F_o_ and F_c_ are the observed and calculated structure factors, respectively; ^d^ A 5% random test set; ^e^ Calculated using Molprobity.

## Abbreviations

MAC	Minimum Agglutination Concentration
MIC	Minimum Inhibition *C*oncentration
PCR	Polymerase Chain Reaction
CD	Circular Dichroism
CRD	Carbohydrate-Recognition Domain
Gal-8	Galectin-8
Gal-8_1-186	The truncated protein of Galectin-8 contain 1 to 186 amino acid
Gal-8 N	N-terminal CRD of Galectin-8
Gal-8 L	“long” Gal-8,with the longest linker peptide
Gal-8 M	“medium” Gal-8, with an intermediate linker region
SDS-PAGE	Sodium Dodecyl Sulfate Polyacrylamide Gel Electropheresis
